# Effects on the pulmonary hemodynamics and gas exchange with a speed modulated right ventricular assist rotary blood pump: a numerical study

**DOI:** 10.1186/s12938-018-0591-4

**Published:** 2018-10-20

**Authors:** Feng Huang, Zhe Gou, Yang Fu, Xiaodong Ruan

**Affiliations:** 1 College of Metrology & Measurement Engineering, China Jiliang University, Xueyuan Road 258, Hangzhou, China; 2 State Key Laboratory of Fluid Power and Mechatronic Systems, Zhejiang University, Hangzhou, China; 3 School of Mechanical and Automotive Engineering, Zhejiang University of Science and Technology, Hangzhou, China

**Keywords:** Speed modulation, Hemodynamics, Pulmonary gas exchange, Rotary blood pump, Right ventricular assist

## Abstract

Rotary blood pumps (RBPs) are the newest generation of ventricular assist devices. Although their continuous flow characteristics have been accepted widely, more and more research has focused on the pulsatile modulation of RBPs in an attempt to provide better perfusion. In this study, we investigated the effects of an axial RBP serving as the right ventricular assist device on pulmonary hemodynamics and gas exchange using a numerical method with a complete cardiovascular model along with airway mechanics and a gas exchange model. The RBP runs in both constant speed and synchronized pulsatile modes using speed modulation. Hemodynamics and airway O_2_ and CO_2_ partial pressures were obtained under normal physiological conditions, and right ventricle failure conditions with or without RBP. Our results showed that the pulsatile mode of the RBP could support right ventricular assist to restore most hemodynamics. Using speed modulation, both pulmonary arterial pressure and flow pulsatility were increased, while there was only very little effect on alveolar O_2_ and CO_2_ partial pressures. This study could provide basic insight into the influence of pulmonary hemodynamics and gas exchange with speed modulated right ventricular assist RBPs, which is concerned when designing their pulsatile control methods.

## Background

Rotary blood pumps (RBPs) have become the most popular ventricular assist device (VAD) due to their numerous advantages [1, 2]. Among the existing RBPs, most are designed to assist the left ventricle because it is subjected to a heavy systemic circulatory load and is more likely to fail than the right side. However, there are still some scenarios in which a right ventricular assist device (RVAD) is required, such as severe right ventricular failure after implantation of a left ventricular assist device (LVAD) [3]. Some studies have focused on the development of RVADs, in particular, in which two up-to-date prototypes are RBPs [2].

Throughout the extensive usage of RBPs, other than the constant speed operation mode, the idea to develop pulsing control methods to make RBPs “beat” more like the natural heart has been put forward [4–9]. The physiological effects of pulsatile versus continuous blood flow have been studied extensively [10–12]. It has been demonstrated that pulsatile flow would not only unload the heart better, but would also not induce the typical “stiffening” of peripheral arteries typically found in a continuous flow state [12]. Besides, pulsatile perfusion has the advantages of causing less vital organ injury and systemic inflammation [10].

Up to now, almost all of the pulsatile operation methods have focused on RBPs used as LVADs. Along with the development of specific RBPs used as RVADs, the same issue also arises if to make them pulsatile. Taking the benefits of the pulsatile perfusion mentioned before into account, and considering the fact that even in the pulmonary vein the flow pulsatility is still much obvious [13], it is desirable to design pulsatile control methods for right ventricular assist RBPs.

Before the control method design, it is necessary to investigate the physiological effect with pulsatile operation of right ventricular assist RBPs. With different purposes, there are several methods that could be used. To investigate a specific part of the cardiovascular system or three-dimensional structures of blood vessels, CFD is thought to be a good tool and adopted by many researchers [14–16]. Apart from CFD, some researchers newly introduced state-space approaches when studying the artery wall [17–19]. These are all effective methods to investigate the effect of pulsatile flow in cardiovascular system. However, when regarding the whole circulation system and two-dimensional hemodynamics, a simple system model could be effective and computational saving, and will be adopted in this study [20].

Generally, the pulsatile operation is realized by rotary speed modulation synchronized with the heartbeat. For LVADs, researchers usually only consider the resultant hemodynamic effects. However, for pulmonary circulatory assist another factor could also be taken into account. There is a unique important function of the pulmonary circulation that it is where oxygen (O_2_) and carbon dioxide (CO_2_) gas exchange take place between the blood in the lungs and the atmosphere. Hence, besides hemodynamics, we are going to also include the effect investigation of pulmonary gas exchange when implementing the pulsatile modulation operation for a right ventricular assist RBP.

In this study, we investigated the effects of a right ventricular assist RBP running in both constant and synchronized pulsatile mode on pulmonary hemodynamics and O_2_ and CO_2_ gas exchange using a numerical method. In addition to the complete lumped parameter cardiovascular model, which includes both systemic and pulmonary circulation, models for airway mechanics and gas exchange for O_2_ and CO_2_ between the lung and the atmosphere were also obtained. Using an RBP model in various speed patterns to assist the failing right ventricle, hemodynamics and airway O_2_ and CO_2_ partial pressures were obtained, revealing the effects of these speed modulations.

## Methods

### Cardiovascular model

**Fig. 1:**
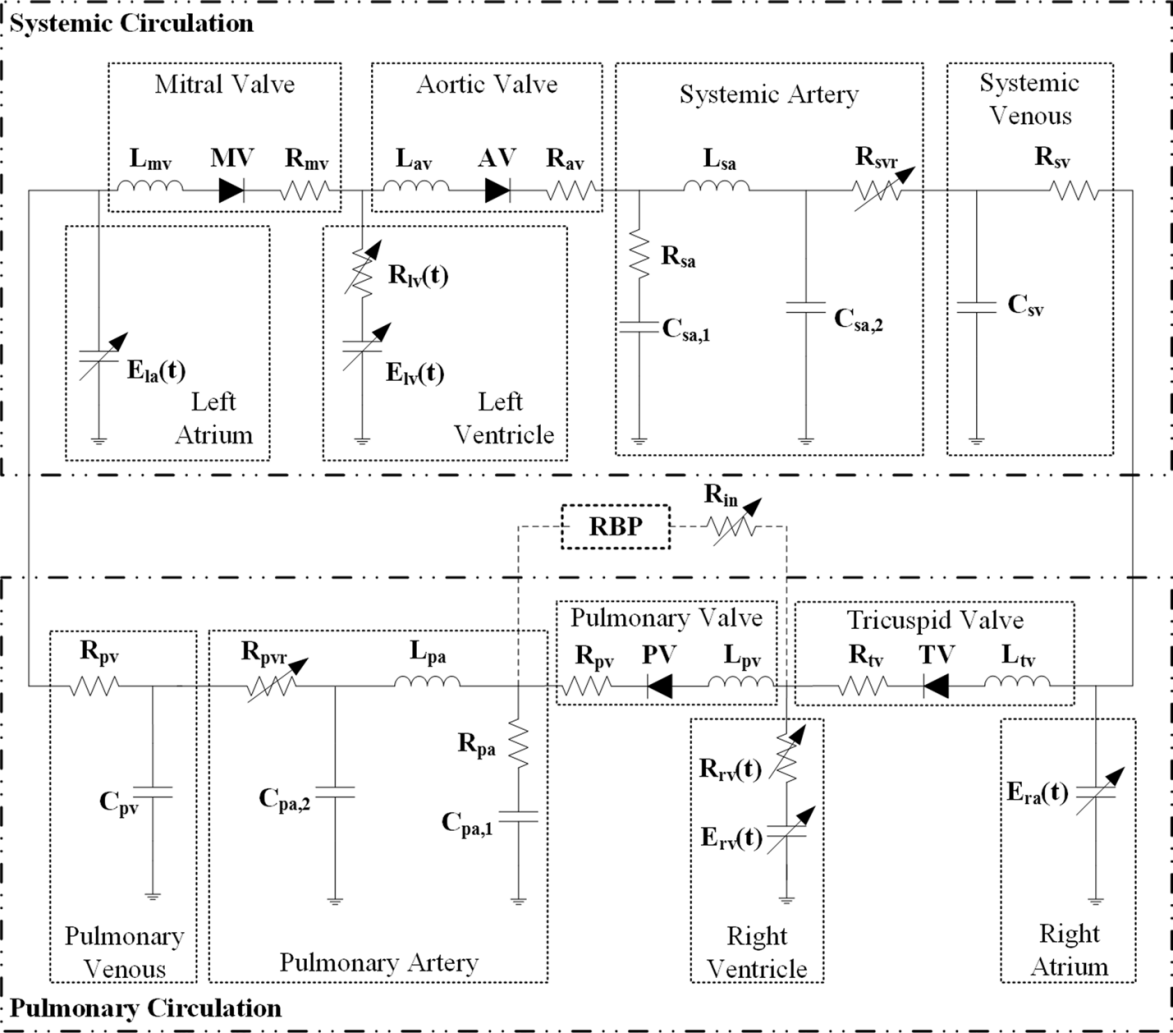
Model of the complete cardiovascular system

To investigate pulmonary hemodynamics and gas exchange with an implanted right RBP, a mathematical model of the complete cardiovascular system, including the systemic and pulmonary circulation, was adopted from a previous study by Colacino et al. [20] (Fig. 1). The four heart chambers are described using the nonlinear time-varying elastance model, with different elastance values between ventricles and atria. In addition, an internal resistance of each ventricle was also included to take into account the energy dissipation during ejection. The heart valve was modeled by an ideal diode representing the one-way function, coupled with resistance and inertance.

The five-elements model was applied to both the systemic and pulmonary arterial systems as parts of the cardiovascular mathematic model. It can reproduce the main arterial characteristics over the entire frequency range of interest [21]. The systemic and pulmonary venous systems are both characterized by resistance and compliance.

### Airway and lung mechanics model

**Fig. 2:**
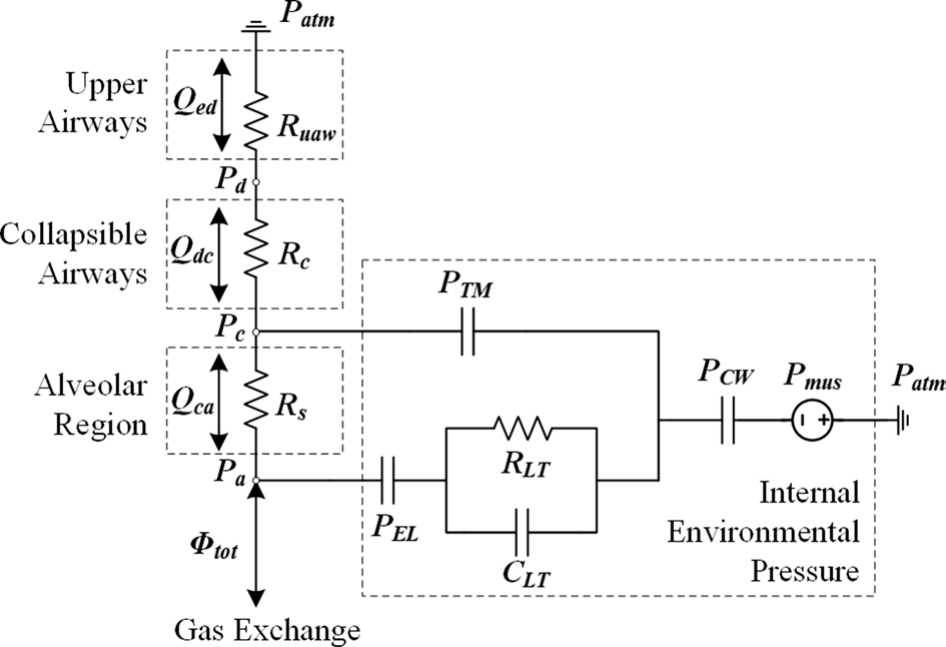
Airway mechanics model

Air from the atmosphere enters the alveoli of the lung, where gas exchange takes place. The airway and lung mechanics model included in this study was adopted from a previous study by Lu et al. [22] (Fig. 2, pneumatic circuit representation). The driving pressure of the air flow is the intrathoracic pleural pressure generated by the respiratory muscles (*P*_*mus*_) and the recoil of the chest wall (*P*_*CW*_), which implies that the frequency of breathing is determined by the intrathoracic pleural pressure in the model. The upper, middle, and small airways are characterized by a nonlinear flow-dependent resistor, a nonlinear collapsible-segment volume-dependent resistance and nonlinear P–V relationship (*P*_*TM*_), and a nonlinear alveolar volume-dependent resistance, respectively [22, 23].

### Gas exchange model

**Fig. 3:**
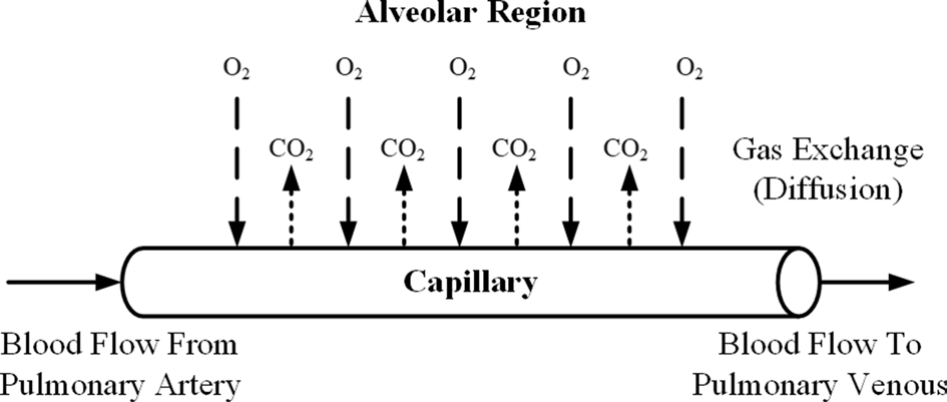
The illustration of the gas exchange across the alveolar–capillary membrane. The blood flow rate is generated by the cardiovascular system model

The gaseous species considered in this study are mainly O_2_ and CO_2_. Gases come from the airway to the alveolar region (Fig. 2) and diffuse across the alveolar–capillary membrane (Fig. 3), where they are thought to equilibrate instantaneously. Specifically, O_2_ is taken up by blood flowing in the capillary, while CO_2_ is removed from the blood. The capillary is considered as a single tube. The relationship between species content and their corresponding equilibrium partial pressures is described by the empirical dissociation curves. The diffusion for a specific gas, which is assumed here to be the only mode of gas transport across the membrane, is characterized by a lumped diffusing capacity. The dynamics of the species concentration in the pulmonary capillary is described by a partial differential equation. For modeling simplicity, some of the same assumptions are also made as detailed by Lu [22] and Liu [23], such that the gaseous content obeys the ideal gas law and blood is characterized as a uniform homogeneous medium. Using the species conservation law, the dynamic partial pressures of O_2_ and CO_2_ in the airways and blood can be described by formulas for both inspiration and expiration processes [22, 23].

### Model of the right ventricular assist RBP

The RBP serving as the right ventricular assistance is connected to the blood circulatory system via cannulation from the right ventricle to the pulmonary artery (Fig. 1). There is a worldwide lack of existing RBPs with hydraulic characteristics designed especially for right ventricle assisting; therefore, there is no ready-to-use model of a right RBP. Here we used the method proposed by Krabatsch et al. [24], which enables the use of an already available LVAD for right ventricular assistance. The main problem of using an LVAD as an RVAD is the excessive flowrate against the much lower pulmonary resistance compared with the systemic resistance, even at the lowest rotary speed. The key point of the proposed method was to add an additional resistance in series with the left ventricular assist RBP in order to decrease the flow delivered into the pulmonary circulation.

In our simulation, the left ventricular assist RBP chosen to act as an RVAD was an axial flow pump reported previously by Choi et al. [25]. The dynamic hydraulic characteristic of the original pump can be described using the following equation [25], in which the pressure head (H) depends on the flow rate (Q), its derivative $\left( {{\dot{\text{Q}}}} \right)$, and the rotary speed (ω).

1 $$ {\text{H}} = a_{0} Q + a_{1} \dot{Q} + a_{2} \omega^{2} $$

where *a*_0_ = − 0.296, *a*_1_ = − 0.027, and *a*_2_ = 0.0000933 are the coefficients identified using dynamic experimental data [25]. In the above equation, the units for the flow rate, pressure head, and rotary speed are mL/s, mmHg, and rad/s, respectively.

**Fig. 4:**
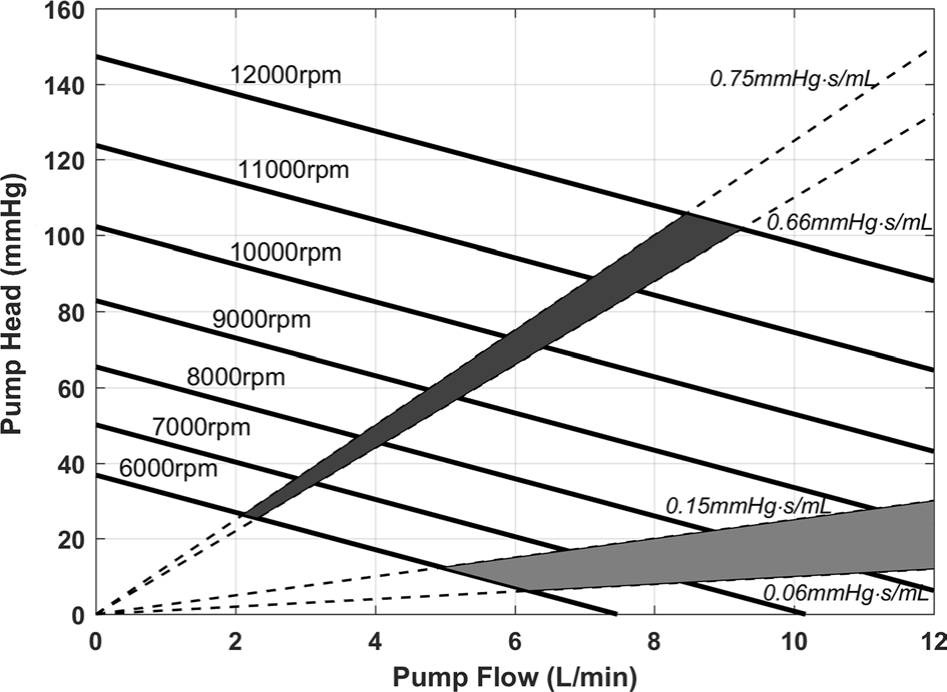
The static H–Q curves and the operational areas of the axial pump before and after the added resistance 0.6 mmHg s/mL. The normal range of the pulmonary resistance is found between 0.06 and 0.15 mmHg s/mL

To adopt the axial pump as the RVAD, an additional resistance (0.6 mmHg s/mL) term was added in the pump model. The corresponding working areas of the axial pump before and after the added resistance on the static H–Q plane can be seen in Fig. 4.

### Computational settings

All of the models were programmed using Simulink/Matlab software (The MathWorks Inc., Natick, MA, USA). The first-order spatial derivative in the gas exchange model was approximated by using a four-point upwind biased formula [26], after which all the equations of the models were ODEs that can be implemented in Simulink. The Runge–Kutta solver and a 10^−3^ s fixed step were chosen in the simulation.

First, simulations without the RVAD were carried out for modeling validation in both normal and right heart failure conditions. Basic hemodynamics and airway gaseous partial pressures were obtained. The right heart failure condition was achieved by setting the right ventricular contractility to 10% of the normal value and the heart rate to 90 bpm. Then the RBP was connected in parallel to the failing right ventricle. Constant rotary speed and sinusoidal modulated speeds were compared. The mean value of the sinusoidal speed profile was the same as the constant speed, which was set to 9000 rpm. The amplitudes of the sine speed profiles were set to 1000, 2000, and 3000 rpm, respectively. The sinusoidal modulated speed was synchronized with the heartbeat, meaning that the pump was running in copulsation mode. During all the simulations, the breathing and gas exchange were always considered normal.

## Results

### Hemodynamics

**Fig. 5:**
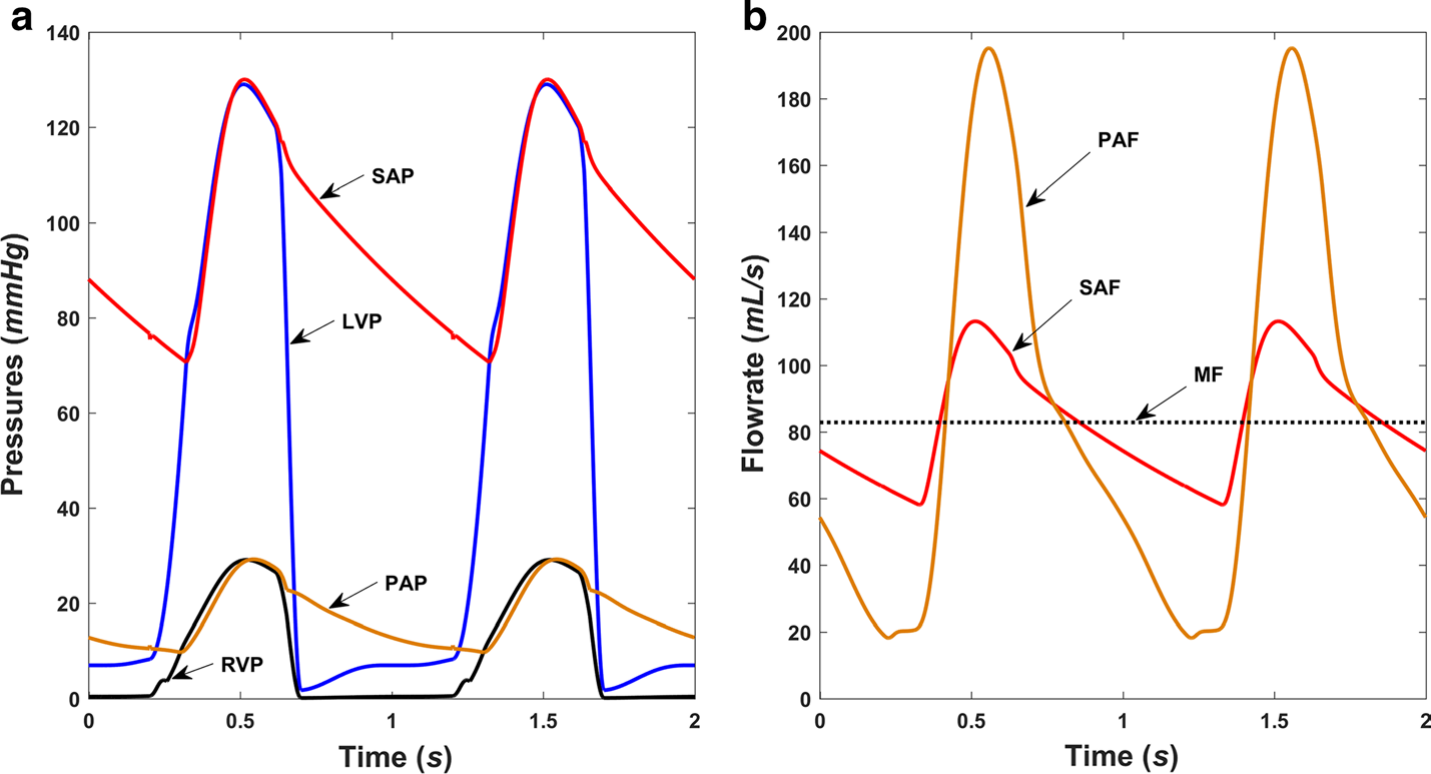
Simulated hemodynamics of the cardiovascular system in normal conditions. **a** Pressure waveforms and **b** flow waveforms. *LVP* left ventricular pressure, *SAP* systemic arterial pressure, *RVP* right ventricular pressure, *PAP* pulmonary arterial pressure, *SAF* systemic arterial flowrate, *PAF* pulmonary arterial flowrate, *MF* mean flowrate of the systemic circulation

**Fig. 6:**
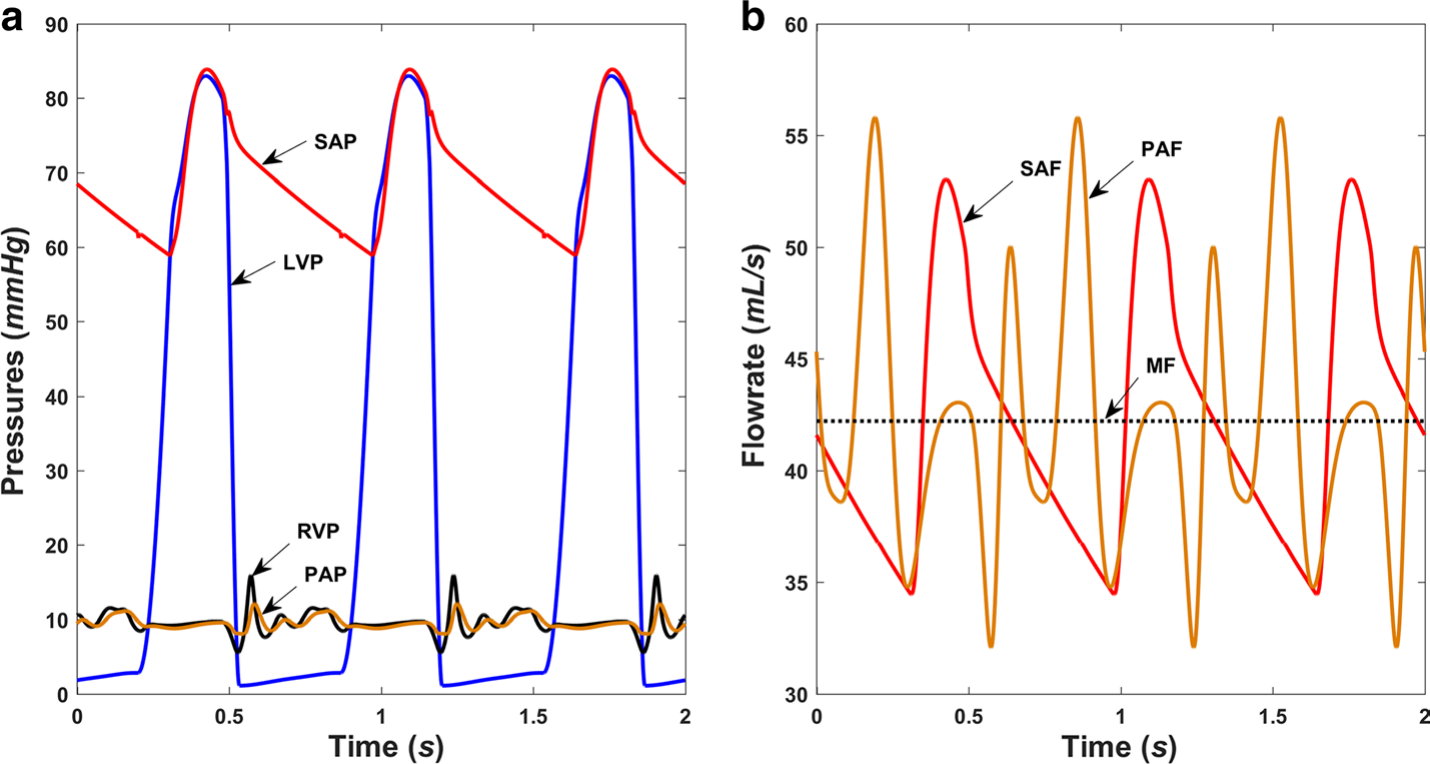
Simulated hemodynamics of the cardiovascular system in right ventricle failure condition. **a** Pressure waveforms and **b** flow waveforms. *LVP* left ventricular pressure, *SAP* systemic arterial pressure, *RVP* right ventricular pressure, *PAP* pulmonary arterial pressure, *SAF* systemic arterial flowrate, *PAF* pulmonary arterial flowrate, *MF* mean flowrate of the systemic circulation

Basic hemodynamics in both normal and right heart failure conditions without the RBP were obtained and are shown in Figs. 5 and 6, respectively. Pressures in normal physiological conditions at the main positions of the blood circulation are depicted in Fig. 5a, while those during right ventricular failure can be seen in Fig. 6a. Normal left ventricular pressure (LVP) ranges from 3 to 129 mmHg, compared with a range of 2–82 mmHg in right heart failure conditions. Systemic arterial pressures (SAP) in the two conditions are 72–129 mmHg and 59–82 mmHg, respectively. The LVP waveform is followed well by the SAP waveform in the systolic period. The corresponding systemic arterial flowrate (SAF) measured at the node R_svr_ in Fig. 1 is also pulsatile, with a range of 58–113 mL/s and a mean value of 83 mL/s for normal conditions, and 34–53 mL/s with a mean value of 42 mL/s for right heart failure conditions (Figs. 5b and 6b). These hemodynamic results are in accordance with those reported in previous studies [20].

Normally, the right ventricular pressure (RVP), which ranges from 0 to 29 mmHg, and the pulmonary arterial pressure (PAP), which ranges from 10 to 29 mmHg, are consistent with physiological values. During right ventricle failure, RVP and PAP fluctuate within a narrow range, and the waveforms becomes disordered (Fig. 6a), as does the pulmonary arterial flowrate (PAF) waveform measured at the node R_pvr_. It is worth noting that the flow pulsatile in the pulmonary circulation is much larger than in the systemic circulation, and both decrease significantly during right heart failure. Another point worth noting is the change in heart period from 60 to 90 bpm between the two figures, which is in accordance with the settings.

### Airway gaseous partial pressure

**Fig. 7:**
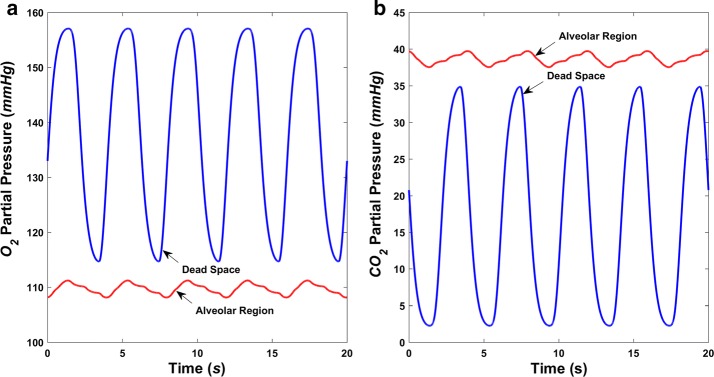
Simulated airway gaseous partial pressure during normal breathing in normal hemodynamic condition. **a** O_2_ partial pressure (PO_2_) in the dead space and alveolar region and **b** CO_2_ partial pressure (PCO_2_) in the dead space and alveolar region

**Fig. 8:**
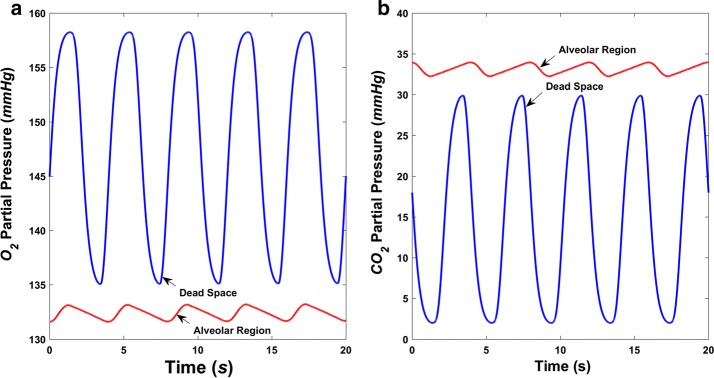
Simulated airway gaseous partial pressure during normal breathing in right ventricle failure condition. **a** O_2_ partial pressure (PO_2_) and **b** CO_2_ partial pressure (PCO_2_) in the dead space and alveolar region

O_2_-enriched air fills into the alveoli during inspiration, where gas exchange takes place, taking away O_2_ and leaving CO_2_, followed by expiration where the air is discharged into the atmosphere again. The variation in airway gas composition in terms of changes in the partial pressures of O_2_ (PO_2_) and CO_2_ (PCO_2_) generated by simulation during this process is depicted in Fig. 7 when the right ventricle is normal, and Fig. 8, when the right ventricle is failing, respectively. The variation of both PO_2_ and PCO_2_ in the up airways (dead space) is much more dramatic than that in the alveolar region. As shown in Fig. 7, alveolar PO_2_ varies from 107 to 113 mmHg and PCO_2_ varies from 37 to 40 mmHg, both of which vary within a narrow range. Not all inhaled air enters the alveoli, and inhaled and residual air mix to create variations in alveolar PO_2_ and PCO_2_ much smaller than those in the dead space. During right ventricle failure, alveolar PO_2_ rises to a range of 131–134 mmHg, which is caused by the decreasing of the volume of oxygen that diffuses into the blood. The total flux of O_2_ across the alveolar–capillary membrane is around 1.7 mL/s, compared with 3.5 mL/s in normal conditions. Meanwhile, alveolar PCO_2_ declines to between 32 and 34 mmHg. It is important to note that breathing function was assumed to be normal during the simulation.

### Effects of speed modulation

In this study, the axial blood pump serving as a RVAD during right ventricular failure was run in constant speed mode and sinusoidal modulated speed mode with three different amplitudes. As mentioned previously, the offset of the sinusoidal speed is the same as the constant speed value, which was 9000 rpm in our simulation.

**Fig. 9:**
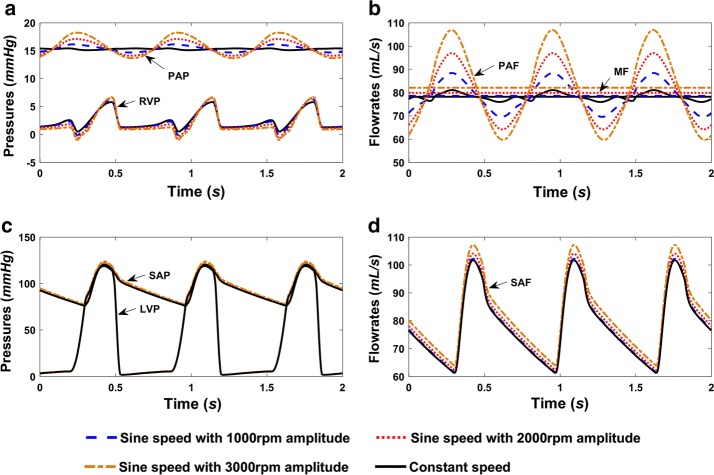
Hemodynamics of the cardiovascular system in right ventricle failure condition with the RVAD assisting in constant and sinusoidal speed modes. **a** Pressure waveforms of the pulmonary circulation; **b** flow waveforms of the pulmonary circulation; **c** pressure waveforms of the systemic circulation; and **d** flow waveforms of the systemic circulation. *LVP* left ventricular pressure, *SAP* systemic arterial pressure, *RVP* right ventricular pressure, *PAP* pulmonary arterial pressure, *SAF* systemic arterial flowrate, *PAF* pulmonary arterial flowrate, *MF* mean flowrate of the systemic circulation

The hemodynamic results generated by the numerical model are shown in Fig. 9. Compared with Fig. 6, it is clear that the PAP increased from 10 mmHg to nearly 15 mmHg with the contribution of the assist pump in all of the running modes. Furthermore, the corresponding PAF fluctuates around a mean value of about 80 mL/s (4.8 L/min), which is about twice as great as without the assist pump in Fig. 6b. With the help of the RVAD, regardless of the mode, the systemic circulatory hemodynamics (LVP, SAP, and SAF) returned to normal values, demonstrating the assisting capacity of the pump. Unlike normal physiological conditions, RVP was much lower than PAP during pump assisting, implying most pump function was contributed by the axial blood pump.

In systemic hemodynamics, the speed modulation of the right RBP has very little effect. As can be seen from Fig. 9c, d, the systemic pressure and flow waveforms with different RBP speed profiles (including constant speed) almost overlap. Although all of the running modes could undertake the right ventricular assisting task, a different influence was found in the pulmonary circulation between them. Along with increasing the sinusoidal modulation amplitude (the amplitude of the constant speed could be regarded as zero), PAP and PAF become more pulsatile (Fig. 9a, b); however, these were still much weaker than the pulsatility in the normal physiological conditions (Fig. 5).

**Fig. 10:**
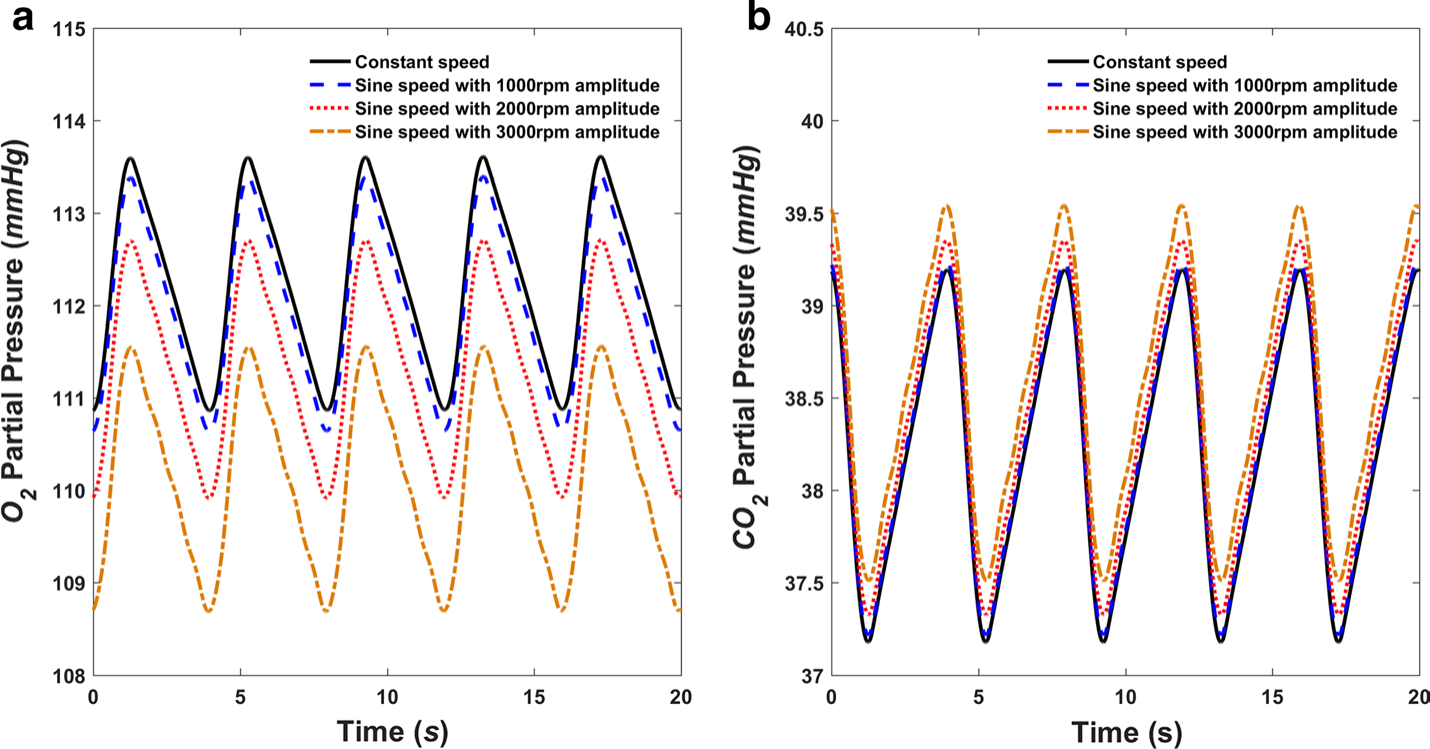
Simulated airway gaseous partial pressures in the alveolar region during normal breathing in right ventricle failure condition with the RVAD assisting in constant and sinusoidal speed modes. **a** O_2_ partial pressure (PO_2_) and **b** CO_2_ partial pressure (PCO_2_)

Figures 7 and 8 show that, when only right ventricle failure occurs but breathing is normal, the alveolar PO_2_ will rise to a relatively high value of more than 131 mmHg, while alveolar PCO_2_ decreases to less than 34 mmHg. After using the assist pump, the alveolar PO_2_ and PCO_2_ are restored to normal ranges, which are around 110 and 38 mmHg, respectively (Fig. 10). With different speed modulations, the alveolar PO_2_ and PCO_2_ have slight differences. The larger the modulation amplitude, the lower the mean value of PO_2_ and the greater the mean value of PCO_2_, whereas the value of change is very small. Along with the increase of amplitude, a trend for the separation between the adjacent PO_2_ waveforms became more obvious (Fig. 10).

## Discussion

Many studies have focused on the pulsatile speed modulation of RBPs as LVADs, whereas there have been few reports on RVADs. Flow pulsation in the pulmonary circulation is remarkable (Fig. 5b). Previous reports have shown the peak flow velocity in pulmonary vein is 52.1 ± 16.4 cm/s [13], and the average pulmonary vein ostium diameter can reach 20 mm [27], which corresponds to a peak volume flow of about 164 ± 51 mL/s. In the present study, the peak flow results agreed with previous studies, side demonstrating the effectiveness of the numerical model. Besides blood transportation, another important function of the pulmonary circulation is gas exchange. Therefore, this study aimed to investigate the effects on both hemodynamics and gas exchange when a right ventricular assist RBP runs in pulsatile mode.

The basic hemodynamics under normal and right ventricle failure conditions generated by the numerical model fit the physiological and pathological results well, demonstrating the effectiveness of the used model. Then sinusoidal speed modulations with different amplitudes under right ventricle failure condition are implemented, revealing that with a sufficient mean rotary speed the hemodynamics could recover back to normal and meanwhile enhance the pulsatility of the pulmonary circulation. The airway gaseous partial pressures also return to normal with the help of the RVAD. Enlarging the modulation amplitude will influence alveolar PO_2_ and PCO_2_ inversely, decreasing PO_2_ and increasing PCO_2_. However, this influence is slight and not as obvious as the hemodynamic effect. Considering the change in mean flowrate (Fig. 9b), this influence might be caused from this mean flow difference. This finding is consistent with a recent report claiming that pulsatile blood flow has only a small to no effect on the gas exchange performance in an oxygenator [28]. Therefore, it implies pulsatile operation using speed modulation would not influence the gas exchange much while could improve the hemodynamics (including pulsatility) enormously. More experimental validations could be carried out further with a breath sensor and a wearable sensor system [29, 30].

As O_2_ diffusion across the alveolar–capillary membrane decreases, alveolar PO_2_ rises during right ventricle failure (Fig. 8). This is due to the low flowrate in the lung, meaning that oxygen diffusing into the blood cannot be taken away immediately. The total flux of O_2_ across the alveolar–capillary membrane decreasing from about 3.5 to nearly 1.7 mL/s could also support this. Lower O_2_ diffusion would mean lower O_2_ partial pressure in the arterial blood after gas exchange, in line with the actual situation in which a patient with right heart failure would have low blood O_2_ partial pressure. Breathing function was regarded as normal during the simulations, whereas generally, heart failure is accompanied by abnormal breathing, such as faster breath frequency. Further models describing the relationship between heart failure and respiratory function are required to improve the numerical simulation.

Another limitation of this study is that it does not include tissue O_2_ consumption and CO_2_ generation model. The initial O_2_ and CO_2_ partial pressures from the venous blood are set as constant values according to the study by Lu et al. [22]. Introducing a tissue O_2_ and CO_2_ exchange model to construct a whole closed-loop cardiovascular–pulmonary tissue model would extend the capability of the numerical study. More physiological conditions, such exercise, can be simulated and it would be beneficial to designing control algorithms of RBPs in use.

The speed modulation method of the RBP adopted in this study was limited to sinusoidal modulation. Only amplitude varied, while the modulation frequency was set at a constant heart rate. Besides, the RBP was run in copulsation mode to enhance the pulsation, and no other modes, such as counterpulsation mode was implemented in this study. To give a more comprehensive understanding of speed modulation of continuous flow RVAD, investigations using more rotary speed modulated waveforms, such as square wave, and more modulation modes, such as counterpulsation mode, and even asynchronous modulation with different heartbeats, could be carried out in the future.

## Conclusions

In this study, we investigated the effects of a right ventricular assist RBP running in both constant and synchronized pulsatile modes on the pulmonary hemodynamics and gas exchange using a numerical method. Basic hemodynamics and airway O_2_ and CO_2_ partial pressures in both normal physiological conditions and during right ventricle failure were obtained. Results showed that the pulsatile run mode of the assist pump was able to recover most hemodynamics to normal levels during right ventricular failure, and that speed modulation could obviously increase the flow and pressure pulsatility in the pulmonary circulation while with only little effect on the pulmonary gas exchange. This study could provide basic insight of the influence of speed modulation of a right ventricular assist RBP when designing pulsatile control algorithms for them.
